# First Report on the Genetic Diversity of Populations of *Gossypium barbadense* L. and *Gossypium hirsutun* L. in the Amazonian Native Communities, Cusco-Peru

**DOI:** 10.3390/plants12040865

**Published:** 2023-02-14

**Authors:** Luis Morales-Aranibar, Francisca Elena Yucra Yucra, Carlos Genaro Morales Aranibar, Manuel Canto Sáenz, Hebert Hernán Soto Gonzales, Jorge González Aguilera, Juan Luis Lazo Álvarez, Alan Mario Zuffo, Fabio Steiner, Rafael Felippe Ratke, Paulo Eduardo Teodoro

**Affiliations:** 1 National Intercultural University of Quillabamba, Cusco 08741, Peru; 2 National University of Moquegua (UNAM), Ilo 18601, Peru; 3 Universidad Nacional Agraria La Molina (UNALM), Lima 012, Peru; 4 Department of Crop Science, State University of Mato Grosso do Sul, Cassilândia 79540-000, MS, Brazil; 5 Peruvian Institute of Cotton of Peru, San Miguel, Lima 15086, Peru; 6 Department of Agronomy, State University of Maranhão, Campus de Balsas, Balsas 65800-000, MA, Brazil; 7 Department of Agronomy, Federal University of Mato Grosso do Sul, Chapadão do Sul 79560-000, MS, Brazil

**Keywords:** cotton, conservation, genetic resources, multivariate analysis, native varieties

## Abstract

The genus *Gossypium* has important ethnobotanical and economic value for Amazonian Native Communities (A.N.C.). However, little research has been undertaken on the distribution and genetic diversity of cotton populations maintained in the Peruvian rainforest. This work aims to present the first report on the genetic diversity of *Gossypium* spp. populations in the A.N.C. of the province of La Convención, Cusco-Peru. The methodology was based on exploring, collecting, identifying, and characterizing the *Gossypium* populations present in the A.N.C. Twenty-six descriptors were evaluated (9 quantitative and 17 qualitative), and with this information, distribution, correlation, and principal component (PC) analyses were carried out. As a result, plants of two species [*G. barbadense* L. (44 samples) and *G. hirsutum* L. (19 samples)], one variety [*G. barbadense* var. *brasiliensis* (75 samples)], and three previously unidentified variations (9 samples) were identified. Altogether, 147 samples were collected. *G. barbadense* var. *brasiliensis*, which was always found in association with other economic crops within an altitude range of 338 to 1086 m, was the most predominant (51%), distributed in eleven A.N.C. and always in small plots (up to 2 ha). *G. barbadense* L. was cultivated between 397 and 1137 m of altitude in eight A.N.C. in plots of up to 3 ha in marginal lands. *G. hirsutum* L., with a smaller distribution (13%), was found between 334 and 497 m of altitude in only three communities; this species is cultivated in marginal areas throughout the year. The variability found for the first two PCs when considering the quantitative and qualitative descriptors was high (74.7%) and moderate (48.2%), respectively. When combining all the descriptors, the analysis showed that the first two PCs accounted for 51.8% of the total variability of the data. The PCs of the two types of data and their combination confirmed that the three populations found were grouped. The nine undefined samples were close to or intermediate between the described ones, showing that these samples may be the result of spontaneous crosses; as such, these samples need to be better evaluated with other tools for further definition. The information obtained shows that in the A.N.C. of Cusco-Peru, there is variability conserved by the inhabitants, who have been able to maintain and use these genotypes, even from their Amazonian indigenous ancestry, and the environment has been able to generate variability among the species, as will be highlighted in future works.

## 1. Introduction

Peru has a high degree of ecological diversity of climates and species. As a result, information is still lacking on the biodiversity of *Gossypium* spp. in some Peruvian Amazonian regions. To date, some species cultivated on the Peruvian coast have been described and found in more than 68 countries [1,2].

In the geographical area of the Amazonian Native Communities (A.N.C.), the distribution of cotton has not been widely studied, although its widespread use is known. Current records on the distribution and loss of *Gossypium* spp. species are geographically limited to the Peruvian coast [2,3]. Amazonian native communities cultivate cotton, such as *G. barbadense*, to maintain their ancestral traditions, e.g., in the production of garments (*cushma* in an Amazonian native language), bags, and blankets). These accessories are used in daily life or are sold in the native communities themselves or at handicraft fairs. The garments are made by hand spinning on handmade looms; they often feature ancestral designs which are in line with traditional practices of valuation and ethnic identity. This tradition attracts national and international tourists [4], compelling the A.N.C. to conserve these cotton genotypes.

Globally, four species of *Gossypium* are of economic importance: *G. herbaceum*, *G. arboreum*, *G. hirsutum* L., and *G. barbadense* L. [1,2]. The fiber in 97% of garments comes from allotetraploid cotton (*G. hirsutum* and *G. barbadense*), which was freely domesticated in the Yucatan Peninsula and northwestern South America [5,6]. Studies on allozyme and diversity using RFLP markers [7] with *G. hirsutum* indicated that there is another center of genetic diversity in the Caribbean, where it is hybridized with *G. barbadense*, a modern form of *G. hirsutum* [8]. Wegier [9] indicated that *G. hirsutum* L. has its origin and genetic variability in Mexico. D’eeckenbrugge and Lacape [10] and Peixoto et al. (2022) agreed that *G. hirsutum* L. is native to Central America. Due to its natural fibers, this plant is the most widely used in the world and is the third largest source of natural vegetable oil [11].

To date, two species of economic importance have been identified in Peru (*G. hirsutum* L. and *G. barbadense* L.); however, there are gaps in scientific information. A complete, updated inventory in Peru to determine the distribution and genetic variability of these species is necessary. *G. barbadense* is considered native to the Peruvian coast [6,12], with primary dispersal in the trans-Andean zone to northern South America, then expanding to Central America, the Caribbean and the Pacific [5]. Modern cultivars of *G. barbadense* originate from Sea Island cotton, which was later improved, giving rise to Egyptian and Pima cotton. Chanco et al. [13] reported that species such as *G. raimondii*, *G. barbadense* L., and the botanical variety *G. barbadense* L. var. *brasiliensis* are endemic to Peru. Recently, the introduction of *G. hirsutum* has been observed in different places. However, few studies have confirmed this. This plant has the potential to threaten native species by competing for geographic space [2].

Cotton is a plant that morphologically varies according to its species and environment [14,15]. Humans have improved the morphologies of cotton species during domestication to improve its characteristics, i.e., to make it more useful for anthropogenic activities [5,16]. *G. hirsutum* and *G. barbadense* differ in numerous characteristics, such as seed dormancy, yield, photoperiod, and fiber quality [17]. Recent advances in cotton genome sequencing [18,19] have made it possible to assess the domestication history of *G. hirsutum* and *G. barbadense*. These studies represented the wild/landrace gene pool from ancestries in geographic centers of origin and/or low-coverage sequencing [5].

Knowing the diversity of cotton is important, as *Gossypium* is a genus that easily hybridizes through genotype–environment interactions (G×E) [20,21]. Diversity studies are important and provide new knowledge that makes it possible to improve and recover native cottonwoods; they also deepen our understanding of how species have adapted to different environments [20,21,22]. In the case of Lower and Upper Urubamba in the province of La Convención-Cusco, hybridization is already occurring, as demonstrated in recent studies [22]. Understanding genetic diversity is very important for the recovery of this germplasm.

The conservation of diversity in situ and in ex situ germplasm banks is essential to retain genetic resources as a part of the heritage and legacy of our ancestors who have cultivated and conserved it [3]. Cotton is generally conserved in wild or domesticated forms, either cultivated in agricultural fields or at the level of orchards and gardens [22]. In Amazonian communities, cotton is being lost, and in many cases, it is being replaced by other crops of economic interest, such as cassava, bananas, and oranges. Climate change, which alters the morpho-productive development of cotton, is another problem facing the crop today [23].

Currently, in Peru, there is a need to generate reliable data on the distribution of *Gossypium* phytogenetic resources in order to determine the diversity of species and their geographic distribution and thus initiate protection and conservation measures [1,9]. In the province of La Convención, it has been shown that all N.C.A. cultivate *Gossypium* spp.; however, to date, little information is available on native and introduced varieties, with no reports having been published on the districts of Echarati and Megantoni. The present research aims to present the first report on the genetic diversity of *Gossypium* spp. in the native Amazonian communities of the Province of La Convención, Cusco-Peru.

## 2. Results

Sampling in the Province of La Convención, Cusco, Peru, showed that cottonwoods of the genus *Gossypium* are distributed in the districts of Echarati (54%) and Megantoni (46%), with both being preserved by native Amazonian communities (Table 1). 

Two species [*G. barbadense* L. (30%) and introduced populations of *G. hirsutum* L. (13%)] and one botanical variety [*G. barbadense* L. var. *brasiliensis* (51%)] were identified and characterized from a total of 147 samples collected in the two districts of La Convención Province, distributed in 12 A.N.C.

Among the collected samples, nine (6%) (Timpia community: five samples; Koribeni: one sample; and Poyentimari: three samples) merit further studies, such as molecular studies, to better describe and differentiate them from other species, because they showed different characteristics from the species identified in the other communities.

### 2.1. Distribution of G. barbadense L.

The native varieties of the species *G. barbadense* L. were distributed in eight Amazonian native communities in the districts of Echarati and Megantoni, from 334 m to 1137 m altitude (Table 1). In the Echarati district, 25 samples (57%) were collected, while in the Megantoni district, 19 samples (43%) were collected for this species.

**Figure 1:**
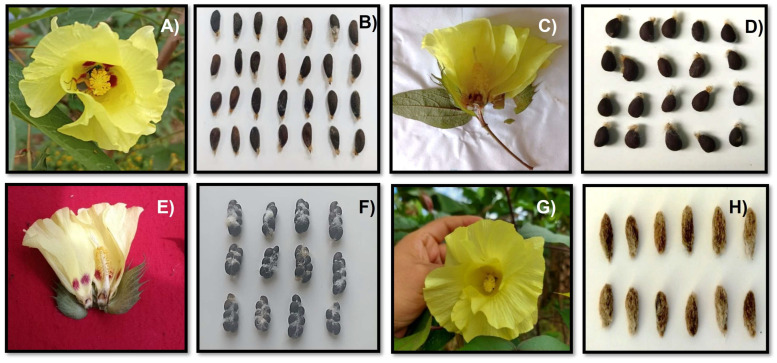
Details of the flowers (**A**,**C**,**E**,**G**) and seeds (**B**,**D**,**F**,**H**) of the species *G. barbadense* L. (**A**–**D**), *G. hirsutum* L. (**G**,**H**) and the variety *G. barbadense* L. var. *brasiliensis (***E**,**F**), Cusco-Peru.

When evaluating the characteristics of each sample, it was observed that for the samples of *G. barbadense* L., the flower was, on average, approximately 7.25 cm long and 3.89 cm wide, and the color of the petals was yellow to narcissus yellow, even cream colored, with the presence of macules ranging from shades of red to intense purple. Spots could be seen with a variation in intensity according to the size of the macula; the larger the spot, the more intense the color (Figure 1A). In some native Amazonian communities, some flowers without maculae were observed, but with characteristics of *G. barbadense* L. (Figure 1C).

*G. barbadense* L. is distributed in three different Amazonian communities in Echarati and five in Megantoni (Table 1). Its distribution is always in places with regular slopes and is associated with crops such as cassava (*Manihot esculenta* Crantz), corn (*Zea mays*), and orange (*Citrus sinensis* (L.) Osbeck), bananas (*Musa* spp.) and beans (*Phaseolus vulgaris* L.). It can be observed on the borders and roadsides and always near the Vilcanota River in the district of Echarati. In these areas, there was evidence of widespread fruiting and adaptation to temperatures ranging from warm to hot, considering the altitude at which the samples were collected (Table 1). In the District of Megantoni, these plants are cultivated in parcels ranging from one to three hectares. They are also found on the edges of farms, on the edges of irrigation ditches and rural roads, and in the gardens of houses. Clay soils are predominant in this region. This species is part of the flora of the banks of the Urubamba River; its presence in the area is due to ancestral ethnobotanical/medicinal practices, as well as for the manufacture of the clothing which is typical of Andean culture.

### 2.2. Distribution of G. barbadense L. var. Brasiliensis

*Brasiliensis* of the species *G. barbadense* L. was the most frequently encountered native variety, with 75 samples collected (Table 1). The variety is distributed between 336 and 1137 m.a.s.l. in the districts of Echarati and Megantoni and is present in 11 native Amazonian communities, as shown in Table 1. The flowers are approximately 7.27 cm long and 4.89 cm wide on average, and the color of the petals ranges from yellow to cream (Figure 1E), with the presence of red to purple macules.

This botanical variety was observed in three communities in the district of Echarati (Table 1), distributed in small cotton plots and always associated with cassava (*Manihot esculenta* Crantz), papaya (*Carica papaya* L.), plantain (*Musa* spp.) and orange (*Citrus sinensis* (L.) Osbeck) crops. The soils where the samples of this species were collected have a high content of organic matter and are always on a regular slope. This species may be observed in the front of some houses and as part of ornamental gardens alongside roads. In the district of Megantoni, it was observed in eight Amazonian native communities (Table 1), distributed in parcels of 1 to 2 ha as a monoculture on flat land with a clay soil texture and few nutrient reserves.

### 2.3. Distribution of the Introduced Species G. hirsutum L.

*G. hirsutum* L. has also been described in the Province of La Convención, located between 364 and 506 m.a.s.l., in the districts of Echarati and Megantoni. The species was found in three native Amazonian communities (Nuevo Mundo, Monte Carmelo, and Poyentimari), as shown in Table 1. It was the least widely distributed: only 19 samples were found (Table 1), whereas for *G. barbadense* L. var. *brasiliensis* and *G. barbadense* L., a total of 75 and 44 samples were found, respectively.

When characterizing the flower of this species, an average length and width of approximately 7.88 cm and 4.45 cm were observed, with yellow to cream-colored petals, without the presence of red to purple macules (Figure 1G). Seeds are loosely arranged and completely covered by olive green linters (Figure 1H), varying from 7 to 9 seeds, ovoid to oval in shape, and mostly dark brown. The fibers are smoke white (Figure 1H).

This species was introduced and is distributed in Echarati, where it can be observed in two native Amazonian communities (Table 1). Its distribution is subspontaneous on hillsides and in small cotton plots. It is always associated with crops such as cassava (*Manihot esculenta* Crantz), papaya (*Carica papaya* L.), plantain (*Musa* spp.) and orange (*Citrus sinensis* (L.) Osbeck). The soils where this species was located had a high organic matter content and were always on a regular slope. Similar to the other described species, it was also observed in the front of some houses or alongside roads as an ornamental plant. In the district of Megantoni, these plants may be found in parcels of less than 1 ha, where they are associated with the cultivation of *Zea mays* on flat ground with a poor clay soil texture near the banks of the lower Urubamba River.

### 2.4. Unidentified Samples of Gossypium spp.

**Figure 2:**
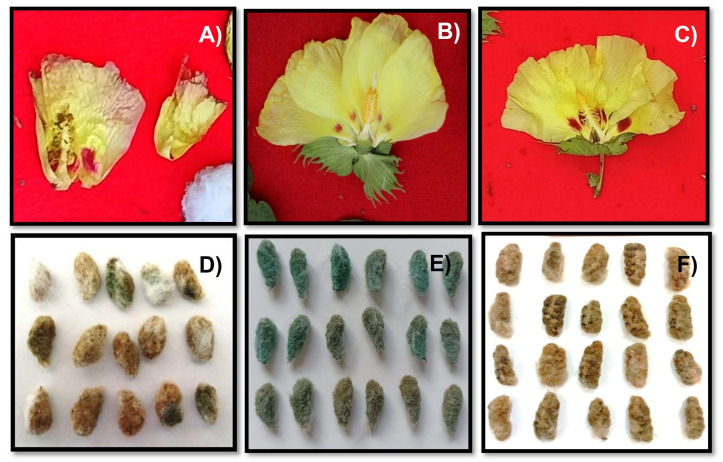
Details of the flowers (**A**–**C**) and seeds (**D**–**F**) of *Gossypium* spp. not classified in this study found in the native communities of Poyentimari (**A**,**D**), Koribeni (**B**,**E**), and Timpia (**C**,**F**), Cusco-Peru.

During the characterization process, the collected samples were classified according to the qualitative characteristics of species described in the literature. As shown in Figure 2, within this process, three groups of samples stood out, as they shared the characteristics of more than one species and, at the same time, differed from the two identified species (Table 1).

Among the three groups of unknown samples, one sample was identified in the Amazonian native community of Poyentimari (G1) in the district of Echarati (Figure 2A,D). A defining characteristic of this sample was that the seeds were completely covered with olive green linter; they were distinct in shape, and the color of their fiber was white with a creamy hue (Figure 2D). The flowers averaged approximately 7.72 cm in length and 4.61 cm in width, and the petal color ranged from pale yellow to whitish cream, with purple macules present (Figure 2A).

A second sample (Figure 2B,E) found in the Koribeni Native Community (G2) was identified as being distinct from the two species described in the sampled areas. The characteristics of this sample were that the flower had purple maculae with green linter throughout the seed (Figure 2E). The flowers were 8.5 cm long by 3.48 cm wide (Figure 2B). The plants were associated with cacao (*Theobroma cacao* L.), masasamba (*Annona muricata* L.), and other medicinal plants. The villagers commented that this species has been in cultivation for more than 10 years; it was only found in one area (Table 1).

Figure 2c,f show the particular characteristics of the third group of cotton samples found in the Timpia community (G3) but not in the Meganto-ni district (Table 1). The flower had purple maculae, measuring 6.90 cm in length by 5.12 cm in width (Figure 2C). The seeds were kidney-shaped and had olive green linters throughout (Figure 2F). This plant was observed on the banks of the lower Urubamba in a monoculture plot in areas larger than 1 ha. The villagers commented that this species has been in cultivation for more than 8 years; it was only found in one area (Table 1).

### 2.5. Quantitative Characterization of the Collected Samples

The results of the analysis of variance performed on the quantitative descriptors obtained when comparing species of Gossypium spp. collected in the province of La Convención are shown in Table 2. There were highly significant differences (*p* < 0.001) between all the species described for all the descriptors used. Low coefficients of variation (<6%) showed the high level of precision of the data obtained when evaluating these field-collected samples.

When the mean values of each of the nine quantitative descriptors were compared, it was observed that there was phenotypic diversity if we considered that within each of them, there were significant differences according to the Tukey’s test (Table 3). For flower length, bract length, leaf length, and leaf width, the highest (8.5 cm, 6.94 cm, 25.48 cm, and 29.8 cm, respectively) and lowest (6.9 cm, 5.74 cm, 12.74 cm, and 15.54 cm, respectively) values corresponded to the unknown samples of groups G2 and G3, respectively (Table 3). For FW and CL, a similar behavior with inversion of positioning was observed, with the highest values for these descriptors being observed in the unknown samples of the G3 group (5.12 cm and 7.30 cm, respectively) and the lowest in the G2 (3.48 cm and 5.84 cm, respectively) group (Table 3). This finding revealed the potential of these unidentified groups for these descriptors. 

For the BW descriptor, the highest and lowest values were obtained for the unknown samples of groups G1 (6.98 cm) and G3 (3.88 cm) (Table 3). For HP, samples from the unknown group G2 (3.58 m) and G1 (3.11 m) stood out, with the highest and lowest values for this descriptor, respectively. These data allowed us to conclude that the differentiation among the described species was evident, possibly indicating new genotypes and the potential of practicing selection in these groups.

For the CW descriptor, the highest values were obtained for the species *G. hirsutum* L. (3.4 cm) and the samples of the unknown group G2 (3.56 cm), which did not differ statistically between them (Table 3). For this same descriptor, the lowest values were obtained for the samples of the unknown group G3 (2.74 cm) and *G. barbadense* L. var. brasiliensis (2.83 cm), with no differences between them.

### 2.6. Correlations of the Quantitative Descriptors Obtained among the Groups Derived from the Collected Samples

**Figure 3:**
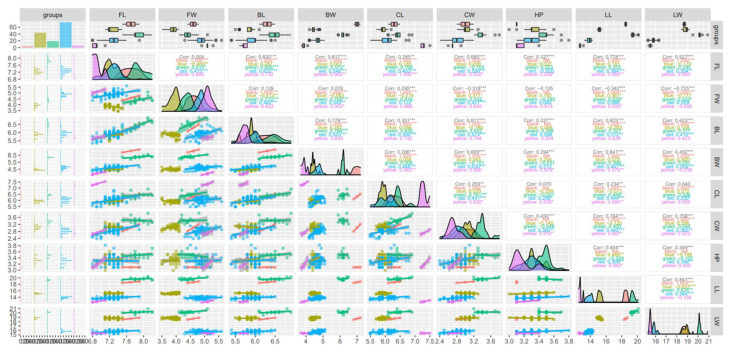
Pearson correlation and scatter plot obtained by comparing nine quantitative descriptors of five cotton groups [Blue (*G. barbadense* L.), red (*G. barbadense* L. var. *brasiliensis*), green (*G. hirsutum* L.), black (unknown group G1) and yellow (unknown group G3)] collected in the province of La Convención, in the districts of Echatari and Megantoni, Cusco-Peru. Flower length (FL, cm), flower width (FW, cm), bracts length (BL, cm), bracts width (BW, cm), capsule length (CL, cm), capsule width (CW, cm), plant height (HP, m), leaf length (LL, cm), and leaf width (LW, cm). Symbols *, **, and *** represent significant differences at 0.5, 0.1, and 0.01% probability, respectively.

Pearson correlations were established among the nine quantitative descriptors when considering five groups of cotton (*G. barbadense* L., *G. barbadense* L. var. *brasiliensis*, *G. hirsutum* L.) and the unknown groups G1 and G3, collected in the province of La Convención, in the districts of Echatari and Megantoni, Cusco-Peru (Figure 3). The data show that there were strong correlations (>70%) between the descriptors FL × BL (83.0%, *p* < 0.001), BW (81.7%, *p* < 0.001), and LL (77.8%, *p* < 0.001), FW × LW (−72.5%, *p* < 0.001), BL and BW (77.9%, *p* < 0.001), BW × LL (84.1%, *p* < 0.001), CW × LL (78.4.7%, *p* < 0.001) and LW (75.8%, *p* < 0.001), and LL × LW (84.1%, *p* < 0.001) (Figure 2). The lowest correlations were observed between FL × FW (0.004%), BW × FW (0.078%), HP × CL (0.07%), and LW × CL (0.04%), all of which were not significant (Figure 3).

Figure 3 also shows the dispersion of the data for each of the groups in the combinations of descriptors (below the main diagonal) and the normality distribution of the data (main diagonal). The first column of Figure 3 shows the distribution of classes within the five groups evaluated in relation to each descriptor evaluated. This column shows that for most of the quantitative descriptors, the population of *G. barbadense* L. var. *brasiliensis* showed the highest number of classes associated with the existing variation within the five groups evaluated according to each descriptor (Figure 3). In the first line of Figure 3, the means are also represented using box-plot graphs, showing that the black group (unknown group G1) showed the highest mean values for most of the descriptors, as described in Table 3, revealing the superiority of this group compared to the two described species and the other unknown group G3. Unknown group G2 was not included in this analysis because it contained only one sample.

### 2.7. Qualitative Characterization of the Collected Samples

**Figure 4:**
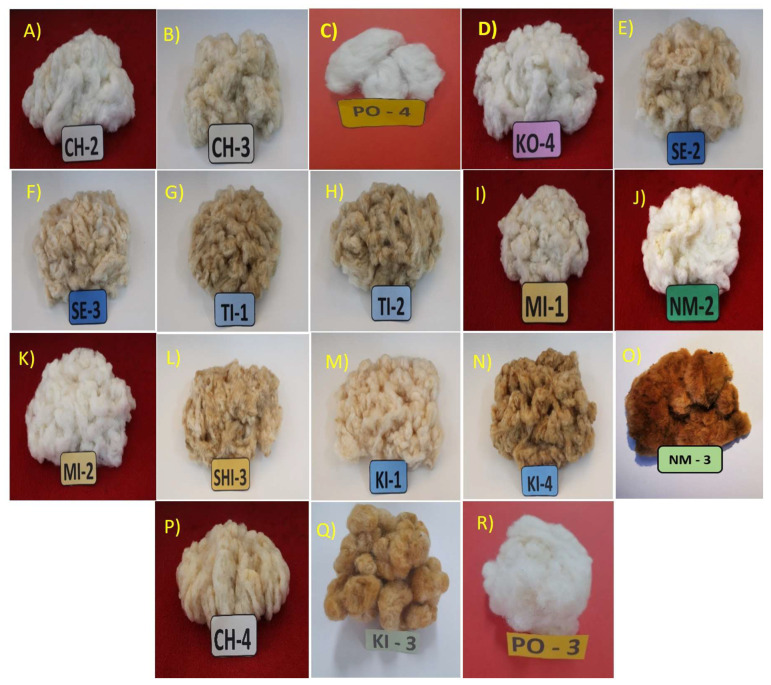
Representation of the colors of the cotton balls obtained in five groups of cotton species [*G. barbadense* L. (**D**,**F**,**I**,**M**,**P**,**Q**), *G. barbadense* L. var. *brasiliensis *(**A**,**B**,**E**,**G**,**H**,**J**,**K**,**L**,**N**,**O**), *G hirsutum* L. (**C**) and the unknown group G1 (**R**)] collected in the province of La Convención, in the districts of Echatari and Megantoni, Cusco-Peru. In (**A**) Floral white; CH-2: Chacopishiato, (**B**) Smoke white; CH-3: Chacopishiato, (**C**) White; PO-3: Poyentimari, (**D**) Anti-flash white; KO-4: Koribeni, (**E**) Pastel brown; SE-2: Sensa, (**F**) Old white; SE-3: Sensa, (**G**) Moccasin; TI-1: Timpia, (**H**) Pearl; TI-2: Timpia, (**I**) Alabaster; MI-1: Miaria, (**J**) Cult; NM-2: New World, (**K**) Chinese white; MI-2: Miaria, (**L**) Champagne; SHI-3; Shivankoreni, (**M**) Bleached almond; KI-1: Kirigueti, (**N**) Camel; KI 4: Kirigueti, (**O**) Brown; NM-3: New World, (**P**) Pink stick; CH-4: Chacopishiato, (**Q**) Brown; KI 3: Kirigueti, and (**R**) Creamy white; PO-3: Poyentimari.

Qualitative characterizations of the samples collected in the field were carried out by evaluating 16 descriptors for the cotton crop; the different classes are described in Table 4. Among the descriptors evaluated, the CE associated with the cotton speck showed the greatest diversity, as 18 different colors were found within it (Table 4). For this descriptor, *G. barbadense* L. and *G. barbadense* L. var. brasiliensis showed 10 and 16 colors, respectively, as a sample of the wide diversity of colors that can be found for these species in Peruvian Amazonian communities (Figure 4C). The species *G. hirsutum* L. showed only two colors [smoke white (Figure 4) and white (Figure 4D)], showing low variability for this descriptor. The nine unknown group members showed only one color for each of the groups, with colors ranging from creamy white in G1 (Figure 4S), white in G2 (Figure 4D), to brownish in G3 (Figure 4R). For this descriptor, Figure 4 shows a sample of the 18 colors found in this study as a representation of the diversity found for this descriptor in Peruvian Amazonian communities.

Another descriptor that showed the second highest number of classes was CB with 6, followed by CSF and CF with 5; NS, CS, DB, CC, and PPL with 4; SS and SLSC with 3; and PSL, PSA, DS, PTC and SL with only 2 classes (Table 4). For the species *G. barbadense* L. and *G. hirsutum* L., as well as the variety *G. barbadense* L. var. *brasiliensis*, variability was observed for most of the qualitative descriptors, except for DS, CS, CC, SL, and PPL, which showed only a single class within each of the groups (Table 4). For the three unidentified groups, the presence of a single class within the different descriptors was more common, except for the descriptors DB, SLSC, and PTC, which showed only two classes for group G1 (Table 4).

### 2.8. Principal Component Analysis (PCA) Using the Qualitative and Quantitative Descriptors

**Figure 5:**
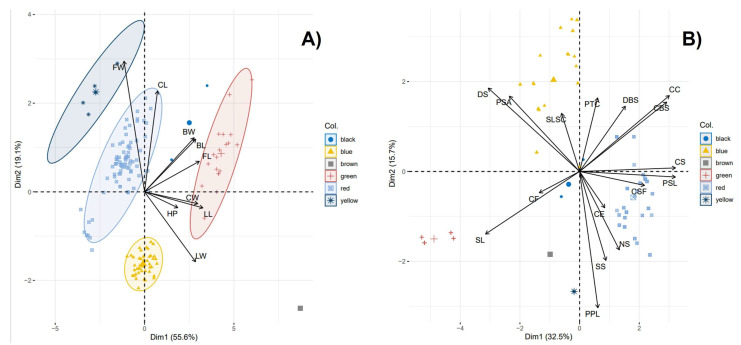
Principal component analysis using nine quantitative (**A**) and 16 qualitative (**B**) descriptors showing the separation of five groups of cotton species [Blue (*G. barbadense* L.), red (*G. barbadense* L. var. *brasiliensis*), green (*G. hirsutum* L.), black (unknown group G1), brown (unknown group G2) and yellow (unknown group G3)] collected in the province of La Convención, in the districts of Echatari and Megantoni, Cusco-Peru. Flower length (FL), flower width (FW), bracts length (BL), bracts width (BW), capsule length (CL), capsule width (CW), height of plant (HP), leaf length (LL), leaf width (LW), color speck (CE), presence of spots on flowers (PSF), color stained flower (CSF), color of flower (CF), position of stigma anthers (PSA), disposal seeds (DS), number of seeds (NS), characteristics of seeds (CS), seed shape (SS), dentate bracterae seeds (DBS), color of bracterae seeds (CBS), shape of longitudinal section capsules (SLSC), characteristics of capsules (CC), prominence of tip capsules (PTC), leaf shape (SL), and presence of pubescence leaf (PPL).

Data from the nine quantitative and 16 qualitative descriptors obtained from characterizing 147 cotton samples collected in Amazonian communities of Peru were used to perform a principal component analysis; the results are shown in Figure 5. When considering the data from the quantitative descriptors (Figure 5A), a high level of variation was observed for the first two components, i.e., describing 74.7% of the variability of the data. For this data set, the a priori characterized samples (groups and samples of unknown origin) were grouped together, mainly for the samples belonging to *G. barbadense* L. (Blue), *G. barbadense* L. var. *brasiliensis* (red) and *G. hirsutum* L. (green) (Figure 5A). This type of data or group formed by sample G2 (brown) was distant from those of the described species, showing that this sample was divergent for these descriptors and deserves attention in future studies. The samples of group G1 (black) were located between the groups formed by *G. barbadense* L. var. brasiliensis (red) and *G. hirsutum* L. (green) or possibly evidenced crosses between the two (Figure 5A). The samples of group G3 (yellow) were located close to the group comprising *G. barbadense* L. var. *brasiliensis* (red); at the same time, this group was closely associated with the descriptor FW, which. among all of the descriptors, was the one with the highest contribution, followed by CL and LW (Figure 5A).

When considering the 16 qualitative descriptors, the PC showed that the first two components retained 48.2% of the total variation in the data (Figure 5B). The analysis showed that the groups formed by the species *G. barbadense* L. (Blue) and *G. hirsutum* L. (Green), as well as the variety *G. barbadense* L. var. *brasiliensis* (red), were distant from each other, indicating that these characteristics determined the differences between these three cotton species (Figure 5b). The species *G. barbadense* L. (Blue) showed a strong association with the descriptors DS, PSA, SLSC, and PTC due to the proximity, magnitude, and direction of the arrows representing each descriptor. The variety *G. barbadense* L. var. *brasiliensis* (red) strongly correlated with the descriptors CS, PSL, CSF, CE, and NS (Figure 5B). The species *G. hirsutum* L. (green) was slightly more distant from the other two varieties described above and was only associated with the descriptor SL (Figure 5B). For this set of qualitative descriptors, the unknown samples of groups G2 and G3 were located close to *G. hirsutum* L. (green) and *G. barbadense* L. var. *brasiliensis* (red) (Figure 5B). On the other hand, the unknown sample of group G1 (black) was located close to the members of *G. barbadense* L. var. *brasiliensis* (red) and *G. barbadense* L. (blue). The location of these unknown groups (G1, G2, and G3), as well as that shown with the quantitative data (Figure 5A), provided evidence that these groups are the product of the hybridization of the described species. However, further studies are required to confirm this.

**Figure 6:**
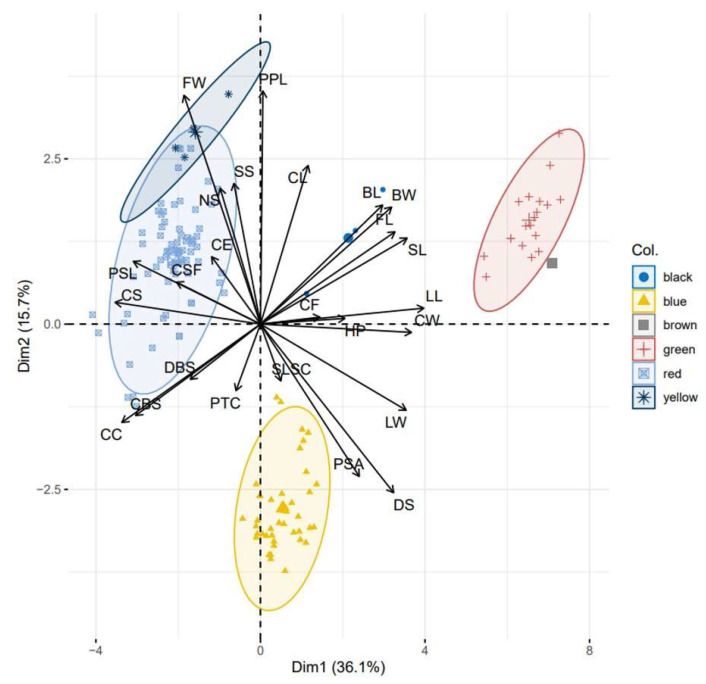
Principal component analysis using the combination of nine quantitative and 16 qualitative descriptors shows five groups of cotton species [blue (*G. barbadense* L.), red (*G. barbadense* L. var. *brasiliensis*), green (*G. hirsutum* L.), black (unknown group G1), brown (unknown group G2) and yellow (unknown group G3)] collected in the province of La Convención, in the districts of Echatari and Megantoni, Cusco-Peru. Flower length (FL), flower width (FW), bracts length (BL), bracts width (BW), capsule length (CL), capsule width (CW), plant height (HP), leaf length (LL), leaf width (LW), color speck (CE), presence of spots on the flower (PSF), color stained flowers (CSF), color of flower (CF), position of stigma anthers (PSA), disposal seeds (DS), number of seeds (NS), characteristics of seeds (CS), seed shape (SS), dentate bracterae seeds (DBS), color of bracterae seeds (CBS), shape of longitudinal section of capsules (SLSC), characteristics of capsules (CC), prominence of tip of capsules (PTC), leaf shape (SL), and presence of pubescence leaves (PPL).

When we combined both types of data (qualitative and quantitative) in the PCs, we found that 51.8% of the variability of the data was retained in the first two components (Figure 6). As observed in Figure 5, the groups associated with the described and unknown *Gossypium* species (G1, G2, and G3) maintained the groupings and the distances between them [*G. barbadense* L. (Blue), *G. barbadense* L. var. *brasiliensis* (red) and *G. hirsutum* (green)] (Figure 6). For the unknown groups, G1 was located between *G. barbadense* L. var. *brasiliensis* (red) and *G. hirsutum* L. (Green), G2 was located close to *G. hirsutum* L. (Green), and G3 was located close to the group formed by the samples of *G. barbadense* L. var. *brasiliensis* (red) (Figure 6).

## 3. Discussion

Currently, there are difficulties in identifying and determining the exact distribution of the cotton populations of the *Gossypium* species, which prevents clearer information about this genus from being made available [6]. There are reviews on the classification of this genus [2,3,24], but there are gaps in the information on the distribution, and the zones are unknown, for example, in the Peruvian Amazon, making it difficult to adequately delimit the species. With this in mind, our work shows the distribution of cottonwood species found and characterized after visiting two districts (Echarati and Megantoni) in the Province of La Convención and finding cottonwoods conserved and distributed in 12 A.N.C. From this fieldwork, 147 samples were identified, and the presence of two species [*G. barbadense* L. (30%) and populations of the introduced species *G. hirsutum* L. (13%)], one botanical variety *G. barbadense* L. var. *brasiliensis* (51%), and in 6% of the total samples collected [community of Timpia (5 samples), Koribeni (1 sample) and Poyentimari (3 samples)], the evaluated characteristics did not allow us to identify the samples at the species level. However, this is the first report of the presence of *G. hirsutum* L. in the Peruvian Amazon. The conservation and distribution levels found for *G. barbadense* L. and *G. hirsutum* L. showed the economic importance they represent for Amazonian communities, owing to the quality of their fibers, as already described by Ozyigit [25] and Ahmed et al. [26].

The genus *Gossypium* comprises more than 50 recognized species distributed in arid and semiarid zones in the tropics and subtropics [27]. Four species were domesticated independently to take advantage of their fibers: *G. arboreum*, *G. herbaceum*, *G. hirsutum*, and *G. barbadense* [2,27]. The description provided by MINAM [2] did not report the presence of *G. hirsutum* L. in the region of Cusco; however, in the regions of Cajamarca, Lambayeque, and Pasco, these species can be found. On the other hand, it is known that *G. hirsutum* L. is an introduced and cultivated species [28]; its origin is Mexico [9], and its fiber is very important for the countries that cultivate it [10,21]. The accounts of the natives living in these localities confirmed the use of this species and showed the importance of these cotton species among indigenous communities. The information obtained in this work shows that there were only incomplete data for the districts of the Cusco region, which underlines the importance of the present study.

*G. barbadense* L. has been reported in 24 regions in Peru [2]. This species, which is thought to originate from northwestern South America, has wild forms and a high level of diversity; hence, the hypothesis is that this species originated in northern Peru and southern Ecuador [6,29]. The importance of this species lies in the quality of its fiber and its improved varieties, destined for spinning and weaving for export and use by native communities [6]. However, Lazo [6] and MINAM [2] reported that this species arrived in the jungle by dispersal in the Amazon, a process that occurred from domesticated forms, probably from what is now Peru, Ecuador, and Colombia, via river networks between Colombia and Peru. This species was found in 8 of the 12 sampled communities, making up 81% of the samples collected. It showed wide diversity for the quantitative (Table 3) and qualitative (Table 4) characteristics of the species in these regions.

According to Lazo [24], *G. barbadense* L. var. *brasiliensis* is of the Amazonian type, with an undetermined origin. It has been noted that it may have spread through the Apure River to the northwest of Colombia, being of interest for the study of the west and east of the Andes. This variety within *G. barbadense* L. was more prevalent (51% of the collected samples) in the districts of Echarate and Megantoni. This result agrees with the studies of Lazo [24], who stated that it was in these same districts that this species has spread the most since its establishment in Amazonia. This variety has managed to adapt to humid tropical conditions and is found in Argentina, Bolivia, Paraguay, northeastern Brazil, the Guianas, and northern South America [2,6,24]. MINAM [30] noted that *G. barbadense* L. var. *brasiliensis* has peculiar characteristics, i.e., its seeds are welded or kidney-shaped, as also observed in our research (Figure 2F). Comments from the inhabitants of the native Amazonian communities indicated that this cotton is the one they wish to cultivate since, at the time of spinning, it is easier to remove, unlike *G. hirsutum* L. and *G. barbadense* L., because its seeds are dispersed in its specks and the fiber is relatively easy to extract. This species is important in the Amazonian communities that cultivate it, constituting a means of production of typical clothing and handicrafts, and therefore, an important source of income [31].

Currently, there is controversy because there is evidence of the introduction of imported yarn from other countries, which means that villagers do not need to grow cotton. This has led to the loss of customs and interest in planting, although these are preserved at present, as indicated in our research (Table 1). Our study also verified the distribution of these species (94% of the collected samples). For the first time, we report the emergence of new genotypes, as shown in the three groups of species not identified among the collected plants (6% of the collected samples). Although these three groups were close to the described species, the PCs showed that they may be the result of natural hybridization between species (Figure 5 and Figure 6), sharing specific characteristics among them while diverging from the described species. This account shows that the diversity of the species, far from being lost, is increasing. Despite their use by local communities, it is necessary to study these unidentified species [2,6,24,30], as they have very good fiber quality while differing from the species that have been found fully identified. This will contribute to reducing the scientific information gap for the described species and the new groups described in this research.

The Cusco region is very popular with tourists who come to Machupichu and stay in the province of Urubamba, which borders the province of La Convención. One of the attractions for tourists is the handicrafts and clothing that the communities produce using the variety of colors that these species provide; our study described 18 different colors (Table 4 and Figure 4). It is speculated that seeds may have been brought by tourists visiting these tourist sites. On the other hand, conversations with the inhabitants of the different Amazonian native communities of Echarati and Megantoni affirmed that many seeds were brought from other departments to improve the quality of the cotton fibers. In addition, others were collected from the wild to select and improve their fiber, without knowing which species they were introducing and the alterations that could occur due to the hybridization that occurs among these species [25,26]. These accounts may explain why 6% of samples did not belong to the two species that our study and previous works have reported for this region [25,26].

As a result of the present work, we found the distribution of two species and one variety of cotton and provided a description of three groups of unidentified samples that have been found in different native Amazonian communities, where hybridizations or speciation may have already occurred. For these three samples, described for the first time here, the application of 9 quantitative and 16 qualitative descriptors associated with principal component analysis allowed us to describe the characteristics of these new groups within the described species. The accounts of the settlers of the different Amazonian native communities affirmed that these new groups have already been there for between 8 and 10 years, although will be verified in later studies. This study lays the foundation for future studies, allowing us to use molecular tools to clarify the origins of these plants and properly place them within the genus *Gossypum*. With this information, we confirm that the diversity of the species for the characterized regions is relevant and that the in situ maintenance and conservation carried out by the Amazonian communities is important, including for investigations such as those proposed in this work, which will allow us to elucidate the distribution of the genus *Gossypium* in this region of Peru.

## 4. Materials and Methods

### 4.1. Study Site and Sampling Method

**Figure 7:**
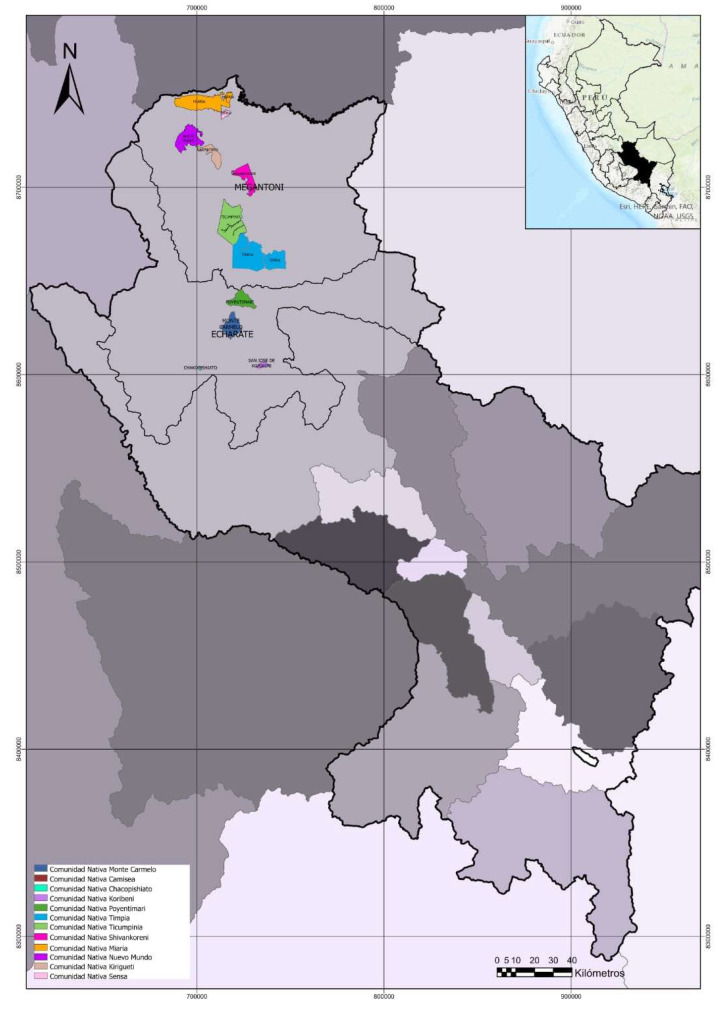
Distribution of collection sites for *Gossypium* spp. in the districts of Echarati and Megantoni, in the Cusco region, in the province of La Convención, Peru.

The study was conducted in the jungle of the province of La Convención of the Cusco Region in Peru, in the Amazonian native communities of Chakopishiato, Poyentimari, Koribeni and Monte Carmelo in the district of Echarati located between the coordinates UTM WGS-84 Zone: 18 South (763082 E and 8587340 N) and in the Miaría, Timpía, Shivankoreni, Kirigueti, Nuevo Mundo, Sensa, Ticumpinía and Camisea communities of the Megantoni district, located between the coordinates UTM WGS-84 Zone: 18 South (724020 E and 8703864 N) Figure 7.

The collections and evaluations were conducted during the period from April to December 2021. Permission was requested from the native Amazonian communities for the collection. Samples of cotton (*Gossypium* spp.) were collected in 12 native Amazonian communities in the districts of Echarati and Megantoni. The type of sampling was nonprobabilistic purposive, by convenience [32]. Within each community, collections were carried out in all places where *Gossypium* spp. was cultivated or found naturally, according to the collection and pressing procedures recommended by MINAM [3].

### 4.2. Qualitative Descriptors Evaluated

Once the samples had been collected, characterization at the species level was carried out according to the following parameters.

In the plants, the following descriptors were evaluated for SPECK: the color of the speck (cotton balls); for FLOWERs: the presence of spots, color of the flower spots, flower color, and position of the stigma in relation to the anthers; for SEEDS: arrangement, number, main characteristics, color, and shape; for BRACTS: types of serration and color; for CAPSULES: shape in longitudinal section, characteristics and prominence of the tip; and finally, for LEAF: shape and presence of pubescence. All these descriptors were evaluated following the recommendations of Manco Céspedes et al. [33] and MINAM [2]. 

All the descriptors were tabulated, and different classes were established for the different species, which allowed us to undertake a descriptive study of the collection sites and the distribution of the main cotton species collected in each locality.

### 4.3. Quantitative Descriptors Assessed

The length and width of the flowers, bracts, capsules, and leaves were determined for all samples. All these measurements were made in cm using a ruler. The height of the plants was also determined in meters with the aid of a ruler. The evaluations followed the recommendations of Manco Céspedes et al. [33] and MINAM [2]. The information generated was tabulated and used in subsequent analyses.

### 4.4. Statistical Analysis

To verify the relationship between the quantitative descriptors and the groups of species, ANOVA was initially performed considering the unbalanced treatments (species), and when significant, the means were compared by Tukey’s test at 1% probability. Using the quantitative data, a scatter plot was made showing the Pearson correlations and the dispersion of each characteristic. The quantitative and qualitative data were used to perform a multivariate analysis using the PCA method to see the overall variability of the two individual datasets and combined for the various groups formed, as well as the trends of the groupings and descriptors. Rbio software [34] and SigmaPlot 10.0^®^ (Systat Software Inc.) were used for the elaboration of the graphs, and R [35] with the “GGally” package was used to generate the correlation plot.

## 5. Conclusions

The present study allowed us to identify two species of *Gossypium spp.* (*G. barbadense* L. and *G. hirsutum* L.) and a botanical variety *G. barbadense* L. var. *brasiliensis*, located in the districts Echarati and Megantoni, along with three groups of samples that could not be identified using the qualitative and quantitative descriptors evaluated. This report contributes to the information that has been presented for this region of the Peruvian Amazon, verifying that there is phenotypic variability for the species *Gossypium* spp. and that conservation efforts are being made by the indigenous people who populate these places. The multivariate analysis tools and the evaluation of different descriptors allowed us to affirm that variability exists within the described species and that the new groups identified may be the result of spontaneous crosses between the described species of *Gossypium* spp.; further studies will be necessary to confirm this hypothesis. The results also show the existence of *G. hirsutum* L. in this zone, and the greatest distribution was observed for the species *G. barbadense* L.

## Figures and Tables

**Table 1:** Distribution of *Gossypium spp.* collected in the province of La Convención in Echatari and Megantoni—Cusco-Peru districts.

District	Community	*G. barbadense* L.	*G. barbadense* L. var. *brasilensis*	*G. hirsutum* L.	Unknown Groups			Total General	Altitude (m)
					G1	G2	G3		
Megantoni	Camisea		3					3	400
	Kirigueti	6	4					10	371–374
	Miaria	1	2					3	334–336
	Nuevo Mundo		6	4				10	364
	Sensa	4	2					6	425–426
	Shivankoreni	7	10					17	397–405
	Ticumpinia	1	2					3	377–382
	Timpia		10				5	15	421–447
Echarati	Chacopishiato	16	20					36	1086–1137
	Koribeni	6	3			1		10	677–746
	Monte Carmelo	3		3				6	428
	Poyentimari		13	12	3			28	497–506
	Total	44	75	19	3	1	5	147	

**Table 2:** *F value* results of the ANOVA performed on quantitative descriptors obtained when comparing species of *Gossypium* spp. collected in the province of La Convención, in the districts of Echatari and Megantoni, Cusco-Peru.

Descriptors	F Value	Significance	CV (%)
Flower-length (FL, cm)	73.57	<0.001	2.29
Flower-width (FW, cm)	146.992	<0.001	4.58
Bracts-length (BL, cm)	44.08	<0.001	3.14
Bracts-width (BW, cm)	375.79	<0.001	3.76
Capsule-length (CL, cm)	53.94	<0.001	3.34
Capsule-width (CW, cm)	54.77	<0.001	5.07
Height-plant (HP, m)	12.40	<0.001	3.6
Leaves-length (LL, cm)	2011.48	<0.001	1.81
Leaves-width (LW, cm)	2960.74	<0.001	1.28

CV: coefficient of variation.

**Table 3:** Comparison of means of quantitative descriptors obtained when characterizing species of *Gossypium* spp. collected in the province of La Convención, in the districts of Echatari and Megantoni, Cusco-Peru.

Gossypium Described		FL	FW	BL	BW	CL	CW	HP	LL	LW
*G. barbadense* L.		7.25 c	3.89 d	5.86 c	4.45 e	5.92 d	3.04 b	3.34 bc	15.18 d	18.81 c
G. *barbadense* L. var. *brasiliensis*		7.27 c	4.89 ab	5.93 c	4.69 d	6.04 c	2.83 c	3.31 cd	13.84 e	15.83 d
*G. hirsutum* L.		7.88 b	4.44 c	6.46 b	6.18 b	6.42 b	3.40 a	3.48 b	19.46 b	20.14 b
*Gossypium* unknown	G1	7.72 b	4.61 bc	6.33 b	6.98 a	6.32 bc	2.99 bc	3.11 d	18.48 c	18.71 c
	G2	8.50 a	3.48 d	6.94 a	5.74 c	5.84 cd	3.56 a	3.58 ab	25.48 a	29.80 a
	G3	6.90 d	5.12 a	5.74 c	3.88 f	7.30 a	2.74 c	3.18 cd	12.74 f	15.54 d
Total		7.35	4.52	5.98	4.84	6.10	3.33	2.97	15.11	17.42

Flower length (FL, cm), flower width (FW, cm), bracts length (BL, cm), bracts width (BW, cm), capsule length (CL, cm), capsule width (CW, cm), plant height (HP, m), leaf length (LL, cm), and leaf width (LW, cm). Letters in columns represent highly significant differences according to the Tukey’s test at 1%.

**Table 4:** Distribution of the number of classes when considering 16 qualitative descriptors obtained when characterizing species of *Gossypium* spp., collected in the province of La Convención, in the districts of Echatari and Megantoni, Cusco-Peru.

***Gossypium* Described**		**CE ^1^**	**PSL**	**CSF**	**CF**	**PSA**	**DS**	**NS**	**CS**
*G. barbadense* L.		10	2	4	3	1	1	2	1
G. *barbadense* L. var. *brasiliensis*		16	1	4	4	2	1	2	1
*G. hirsutum* L.		2	1	1	2	1	1	1	1
*Gossypium* unknown	G1	1	1	1	1	1	1	1	1
	G2	1	1	1	1	1	1	1	1
	G3	1	1	1	1	1	1	1	1
*Total*		19	2	5	5	2	2	4	4
***Gossypium* Described**		**SS**	**DB**	**CB**	**SLSC**	**CC**	**PTC**	**SL**	**PPL**
*G. barbadense* L.		2	3	2	2	1	2	1	1
G. *barbadense* L. var. *brasiliensis*		2	4	2	3	1	2	1	1
*G. hirsutum* L.		2	1	2	2	1	2	1	1
*Gossypium* unknown	G1	1	2	1	2	1	2	1	1
	G2	1	1	1	1	1	1	1	1
	G3	1	1	1	1	1	1	1	1
*Total*		3	4	6	3	4	2	2	4

^1^ Color speck (CE), presence of spots on flowers (PSF), color stained flowers (CSF), color of flowers (CF), position of stigma anthers (PSA), disposal seeds (DS), number of seeds (NS), characteristics of seeds (CS), seed shape (SS), dentate bracterae (DB), color bracterae (CB), shape longitudinal section capsules (SLSC), characteristics of capsules (CC), prominence of tip of capsules (PTC), leaf shape (SL), presence of pubescence of leaf (PPL).

## Data Availability

Not applicable.

## References

[1] López A., López S., Gil E., Caicedo E., Mendoza E. (2018). Characterization of fruits; seeds and fibers of *Gossypium barbadense* “algodón Pardo”. Sciéndo.

[2] MINAM—Ministerio del Ambiente del Perú (2020). Línea de Base de la Diversidad del Algodón Peruano con Fines de Bioseguridad.

[3] MINAM—Ministerio del Ambiente del Perú (2014). Colecta, Elaboración de Mapas de Distribución y Estudio Socioeconómico de la Diversidad del Algodón Nativo.

[4] Rojas I., Cuzquen C., Delgado G. (2014). In vitro clonal propagation rooting of native cotton (*Gossypium barbadense*) cuttings. Rev. Acta Agron..

[5] Yuan D., Grover C.E., Hu G., Pan M., Miller E.R., Conover J.L., Hunt S.P., Udall J.A., Wendel J.F. (2021). Parallel and Intertwining Threads of Domestication in Allopolyploid Cotton. Adv. Sci..

[6] Lazo J. (1990). Articles. Instituto Peruano del Algodón. https://www.ipaperu.org/descarga/ARTICULOS.pdf.

[7] Brubaker C.L., Wendel J.F. (1994). Reevaluating the origin of domesticated cotton (*Gossypium hirsutum*, Malvaceae) using nuclear restriction fragment length polymorphisms (RFLPs). Am. J. Bot..

[8] Stewart J.M., Oosterhuis D., Heitholt J.J., Mauney J.R. (2009). Physiology of Cotton.

[9] Briuolo A.L.W. (2013). Diversidad Genética y Conservación de *Gossypium hirsutum* Silvestre y Cultivado en México. Ph.D. Thesis.

[10] D’eeckenbrugge G.C., Lacape J.M. (2014). Distribution and differentiation of wild, feral, and cultivated populations of perennial upland cotton (*Gossypium hirsutum* L.) in Mesoamerica and the Caribbean. PLoS ONE.

[11] FAOSTAT. http://faostat.fao.org/.

[12] Westengen O.T., Huaman Z., Heun M. (2005). Genetic diversity and geographic pattern in early South American cotton domestication. Theor. Appl. Genet..

[13] Chanco M., León B., Sánchez I. (2006). Malvaceae endémicas del Perú. Rev. Peru. Biol..

[14] López S., Gil A. (2017). Phenology of *Gossypium raimondii* Ulbrich “native cotton” of green fiber. Sci. Agropecu..

[15] López Medina S.E., Mostacero León J., Quijano Jara C.H., Gil Rivero A.E., Rabanal Che León M. (2019). Caracterización Del Fruto; Semilla Y Fibra De algodón Silvestre (*Gossypium raimondii*). Cienc. Tecnol. Agropecu..

[16] Smith C.W., Cothren J.T. (1999). Cotton: Origin, History, Technology, and Production.

[17] Gross B.L., Strasburg J.L. (2010). Cotton domestication: Dramatic changes in a single cell. BMC Biol..

[18] Li F., Fan G., Lu C., Xiao G., Zou C., Kohel R.J., Yu S. (2015). Genome sequence of cultivated Upland cotton (*Gossypium hirsutum* TM-1) provides insights into genome evolution. Nat. Biotechnol..

[19] Zhao Q., Feng Q., Lu H., Li Y., Wang A., Tian Q., Huang X. (2018). Pangenome analysis highlights the extent of genomic variation in cultivated and wild rice. Nat. Genet..

[20] Li Y., Suontama M., Burdon R.D., Dungey H.S. (2017). Genotype by environment interactions in forest tree breeding: Review of methodology and perspectives on research and application. Tree Genet. Genomes.

[21] Peixoto M.A., Malikouski R.G., Nascimento E.F.D., Schuster A., Farias F.J.C., Carvalho L.P., Teodoro P.E., Bhering L.L. (2022). Genotype plus genotype by-environment interaction biplot and genetic diversity analyses on multi-environment trials data of yield and technological traits of cotton cultivars. Ciência Rural.

[22] Hinze L.L., Gazave E., Gore M.A., Fang D.D., Scheffler B.E., Yu J.Z., Jones D.C., Frelichowski J., Percy R.G. (2016). Genetic diversity of the two commercial tetraploid cotton species in the Gossypium diversity reference set. J. Hered..

[23] Wang C., Tang Y., Chen J. (2016). Plant phenological synchrony increases under rapid within-spring warming. Sci. Rep..

[24] Lazo J. (2012). Evolución del algodón *Gossypium barbadense* L. en el Perú y en el continente. Mundo Text..

[25] Ozyigit I.I. (2009). In vitro shoot development from three different nodes of cotton (*Gossypium hirsutum* L.). Not. Bot. Horti Agrobot..

[26] Ahmed H.A., Hajyzadeh M., Barpete S., Ozcan S. (2014). In vitro plant regeneration of Iraqi cotton (*Gossypium hirsutum* L.) cultivars through embryonic axis. J. Biotechnol. Res. Cent..

[27] Delgado-Paredes G.E., Vásquez-Díaz C., Esquerre-Ibáñez B., Huamán-Mera A., Rojas-Idrogo C. (2021). Germplasm collection, in vitro clonal propagation, seed viability and vulnerability of ancient peruvian cotton (*Gossypium barbadense* L.). Pak. J. Bot..

[28] Li L., Zhang C., Huang J., Liu Q., Wei H., Wang H., Yu S. (2021). Genomic analyses reveal the genetic basis of early maturity and identification of loci and candidate genes in upland cotton (*Gossypium hirsutum* L.). Plant Biotechnol. J..

[29] Wendel J.F., Grover C.E., Fang D.D., Percy R.G. (2015). Taxonomy and evolution of the cotton genus; Gossypium. Cotton.

[30] MINAM—Ministerio del Ambiente del Perú (2017). Elaboración del Mapa, Análisis Socioeconómico, y de Organismos y Microorganismos de aire y Suelo. Lineamientos para la Conservación de la Diversidad Genética de la Especie. Informe Final de la Consultoría.

[31] Brozynska M., Furtado A., Henry R.J. (2016). Genomics of crop wild relatives: Expanding the gene pool for crop improvement. Plant Biotechnol. J..

[32] Otzen T., Manterola C. (2017). Sampling Techniques on a Population Study. Int. J. Morphol..

[33] Manco Céspedes E.I., Chanamé Upay J., Arévalo Garazatúa G.M., Mamani Huarachi W.V., Hinostroza García L.D.R., Garay Duran N.H., Lindo Seminario D.E., Vasquez Oroya J., García Serquén A.L. (2022). Descriptores para Algodón Peruano (Gossypium barbadense L.).

[34] Bhering L.L. (2017). Rbio: A Tool for Biometric and Statistical Analysis Using the R Platform. Crop Breed. Appl. Biotechnol..

[35] R Core Team (2014). R: A Language and Environment for Statistical Computing.

